# Combined Spinal-Epidural Anaesthesia for Caesarean Delivery in Takayasu’s Arteritis: A Viable Alternative

**DOI:** 10.7759/cureus.12459

**Published:** 2021-01-03

**Authors:** Koshy Varghese, Amlan Swain, Seelora Sahu, Priyabrata Mohanty, Rajiv Shukla

**Affiliations:** 1 Anaesthesiology, Tata Main Hospital, Jamshedpur, IND

**Keywords:** takayasu arteritis, ceasearean section, vasculitis, anaesthesia

## Abstract

Takayasu's arteritis (TA), also known as “pulseless disease”, is a nonspecific inflammatory arteritis of large and medium caliber arteries of unknown aetiology with a predilection for young women of childbearing age. Although the evolution of the disease is not affected during pregnancy, it can result in uncontrolled hypertension, multiple organ dysfunction, and stenosis that hinder regional blood flow. Associated pregnancy, therefore, poses an increased risk to the mother and foetus due to the many cardiovascular complications that can occur in the course of the disease, making anaesthesia for caesarean delivery especially challenging to the anaesthesiologist. We report the successful anaesthetic management of a case of TA undergoing caesarean section in view of a previous caesarean delivery. We also engage in a brief review of the related literature.

## Introduction

Takayasu's arteritis (TA) is a granulomatous pan-endarteritis of unknown aetiology, with a predilection for Asian women in the reproductive age group, culminating in impaired perfusion of the major organ systems [1,2]. Although pregnancy does not affect the progression of the disease per se, the effects of the multi-faceted pathogenic processes of Takayasu disease are associated with serious cardiovascular implications [3]. In this report, we present the anaesthetic management of a patient of TA scheduled for caesarean section. We obtained the requisite consent to carry out the study and publish its findings.

## Case presentation

A 33-year-old pregnant lady, having TA with grade 2 hypertensive retinopathy and hypothyroidism on medications under regular antenatal care and an uneventful pregnancy, was admitted to our hospital for elective caesarean section. Significant physical findings included her left carotid pulse being impalpable, left radial and brachial pulse being feebly palpable, and blood pressure variance in the limbs.

A 24-hour ambulatory blood pressure monitoring revealed values slotting our patient into the high normal category according to the European Society of Cardiology criteria [4]. The 12-lead ECG and 2D-echocardiography were essentially normal (good left ventricular systolic function having 65% ejection fraction, no right ventricular dysfunction) except for moderate pulmonary arterial (PA) hypertension (estimated PA pressure of 50 mmHg). Apart from anaemia [Haemoglobin (Hb) of 9.2 mg%], her laboratory parameters were within normal limits.

An elective caesarean section was planned in view of her previous caesarean status (emergency caesarean section done under neuraxial block five years back without a diagnosis of TA), and her anaesthesia risk was considered to be acceptable with an American Society of Anesthesiologists (ASA) III physical status risk. Our anaesthetic plan of combined spinal-epidural anaesthesia (CSEA) for the caesarean section was explained to the patient and her relatives, and the risks on account of pregnancy and concomitant TA were clearly mentioned. She was advised to remain on fasting for surgery, and appropriate premedication, including her thyroxine supplementation as well as antihypertensive medications, was administered.

ASA standard monitoring was established on the day of surgery and the baseline parameters were noted [heart rate of 90 bpm, blood pressure of 155/98 mmHg right arm, 132/93 mmHg left arm, 116/81 mmHg right thigh, 111/63 mmHg left thigh, and oxygen saturation (SpO_2_) of 99% on room air on both upper and lower extremities of the left side]. The right arm was selected for non-invasive blood pressure monitoring and the pulse oximetry was used on the left hand. Adequate intravenous access (18 G cannula) was secured, crystalloid co-loading was commenced, and appropriate antibiotic prophylaxis was administered. CSEA was instituted using the loss of resistance (LOR) technique in the sitting position at the L4-5 vertebral interspace. An epidural catheter was placed 4 cms into the epidural space, following which subarachnoid block (SAB) was administered with 25 G Quinkie needle and 2 ml of 0.5% hyperbaric bupivacaine deposited in the subarachnoid space. Additionally, 3 ml of 0.25% bupivacaine was given via the epidural catheter immediately after placing the patient in the supine position, to achieve requisite surgical anaesthesia. Caesarean section was commenced and the surgery was completed in 47 minutes. The intraoperative course, both surgical and anaesthetic, was mostly uneventful except for a single episode of mild hypotension, which was managed with fluid supplementation and a single bolus of vasopressor (mephentermine 6 mg). Her postoperative course was also uneventful and included postoperative pain management by epidural top-ups with 0.0625% bupivacaine.

## Discussion

TA is an autoimmune disease that was first described by Mikito Takayasu, which predominantly afflicts oriental women (8:1) and has a peak incidence during the second and third decades of life [5]. While the aetiology of this distinct large vessel vasculitis is primarily idiopathic, an interplay of immune dysregulation, genetic predisposition in families, and a role of sex hormones appear to be the most plausible explanation [2,5-6]. This arteritis of major vessels like the aorta, the pulmonary artery, and their branches results in stenotic occlusion as well as dilation with aneurysm formation in the affected vasculature [7-9]. The symptomatology complex of these patients shows high variation and ranges from fever, easy fatigability, and gradual emaciation to serious conditions such as cardiac failure and haemoptysis. Several classification systems for TA are described in the literature. They are based either on the anatomical location of the lesion or the existence of major complications such as hypertension, retinopathy, aneurysms, and aortic insufficiency (Ishikawa classification) [10,11]. The presence of retinopathy, aortic regurgitation, and aneurysm formation signal poor prognosis (heart failure, myocardial infarction, and stroke) [4]. The fact that our patient had hypertension albeit controlled and grade II retinopathy (Group III Ishikawa classification) with an absent left carotid artery pulsation was a cause of concern [6].

Preoperative workup should include an assessment of end-organ damage due to arteritis as well as a comprehensive overview of biochemical and radiological changes. Our patient had evidence of stenotic occlusion of the left brachial artery; otherwise, she had normal renal function tests, no demonstrable aneurysmal lesion, and did not manifest other major systemic symptoms. We resorted to routine non-invasive blood pressure monitoring since complications of arterial cannulation are common in patients with peripheral vasculopathies. The classic indications of instituting perioperative invasive blood pressure monitoring include subjects with unmeasurable non-invasive blood pressure, prolonged surgeries, and persistently uncontrolled blood pressure [12].

General anaesthesia, as well as different combinations of regional anaesthesia, have been variously described for caesarean section in patients with TA with reasonable success. However, both general anaesthesia, as well as the more common forms of central neuraxial blockade (SAB or epidural anaesthesia), can reduce organ perfusion significantly. Both modalities hence tend to worsen the existing TA-induced ischemia and have also been associated with a higher rate of perioperative morbidity and mortality. On the other end of the haemodynamic spectrum, general anaesthesia in caesarean sections can also trigger a severe hypertensive response due to inadequate anaesthetic depth during rapid sequence intubation (RSI), till baby delivery or awake extubation [12]. Anterior and posterior circulation stroke (infarction or haemorrhage) leading to visual loss as well as an ocular ischaemic syndrome resulting from hypoperfusion, especially if there is severe occlusion of carotid arteries, has been reported in patients of TA under general anaesthesia [13]. Head and neck positioning under general anaesthesia, in the absence of left carotid pulsation, could have precipitated an ocular ischaemic syndrome in our case. In addition, head extension during laryngoscopy can have potentially deleterious effects on cerebral perfusion while positioning can worsen limb ischemia [7].

In contrast, better haemodynamic control and continuous neurological assessment in an awake patient is a desirable feature of regional anaesthesia, which favoured its choice in our case. Epidural anaesthesia, SAB, and CSEA using low doses of local anaesthetic with or without additives have all been successfully reported in cases of TA. The slow onset of a sympathetic block with a lesser probability of sudden haemodynamic perturbations and hence a better regulation of fluid administration is a desirable feature of epidural anaesthesia besides being a reliable means of adequate postoperative analgesia [14,15]. SAB is technically easier to perform, results in a reliable dense block, and while earlier investigators strictly debunked its use in patients with TA, more recent evidence has shown it to be safe in pregnant patients with controlled haemodynamic parameters [16,17]. Low-dose CSEA overcomes the disadvantage of a patchy epidural or the undesirable haemodynamic fluctuations of SAB and was used successfully in our case with 2 ml of 0.5% hyperbaric bupivacaine heavy in the intrathecal space (reported to be safe by Kathrivel et al.) and then extending the block effect using minimal aliquots (3 ml) of bupivacaine 0.25% in the epidural space to achieve adequate surgical anaesthesia with acceptable haemodynamic alterations, which responded to standard fluid and vasopressor administration [12].

In TA with severe blood pressure discrepancy between the upper and lower limbs (difference of more than 10 mmHg between upper limbs and 20 mmHg between lower limbs), neuraxial anaesthesia is best avoided as it compromises regional blood flow [18]. Our patient exhibited no such discrepancy and this along with a well-controlled blood pressure tilted the choice of anaesthetic technique in favour of low-dose CSEA, even though the patient fell in Group 3 of the Ishikawa classification. The fact that we had an awake, cooperative patient was very reassuring in terms of preserved cerebral perfusion and adequate perioperative urine output (neuraxial blocks do not reduce renal perfusion) further vindicated our choice of anaesthesia technique [18].

Meticulous attention to perioperative fluid and vasopressor administration is paramount in patients with TA because of propensity to cardiac dysfunction and ensuing pulmonary edema [4,19]. Along with the choice of anaesthetic technique, we achieved optimal intraoperative fluid balance and haemodynamics by co-loading the patient, restricting fluid (500 ml of crystalloid), and limiting vasopressor (mephentermine 6 mg only once in our case) use. The use of vasopressors in TA has been associated with dangerous blood pressure elevations and hence should be avoided; no such event was witnessed in our patient [20]. At the same time, hypotension should be avoided in patients with pulmonary arterial hypertension (PAH) as it can compromise the right ventricular perfusion [20].

Pain can also cause unwanted haemodynamic fluctuations or worsen PAH in the postoperative period, and postoperative epidural top-ups with low-dose bupivacaine kept our patient pain-free. These along with the sequential introduction of her regular antihypertensive medications as per her haemodynamic measurements kept her blood pressure controlled in the immediate postoperative period, which was again very critical for the overall outcome.

## Conclusions

In TA with pregnancy, the anaesthetic considerations need to be decided on a case-to-case basis after an in-depth analysis to decide on a safer technique. In this index case, CSEA resulted in a safe patient outcome, and we believe it can be explored as a viable option for caesarean sections in TA.
